# Fluid intelligence and naturalistic task impairments after focal brain lesions

**DOI:** 10.1016/j.cortex.2021.09.020

**Published:** 2022-01

**Authors:** Verity Smith, Clara Pinasco, Jascha Achterberg, Daniel J. Mitchell, Tilak Das, Maria Roca, John Duncan

**Affiliations:** a MRC Cognition and Brain Sciences Unit, University of Cambridge, Cambridge, UK; b Institute of Translational and Cognitive Neuroscience (INCyT), INECO Foundation, Favaloro University, Buenos Aires, Argentina; c Department of Radiology, Addenbrooke's Hospital, Cambridge University Hospitals, Cambridge, UK; d National Scientific and Technical Research Council (CONICET), Buenos Aires, Argentina; e Department of Experimental Psychology, University of Oxford, Oxford, UK

**Keywords:** Executive function, Naturalistic tasks, Fluid intelligence, Default mode network

## Abstract

Classical executive tasks, such as Wisconsin card-sorting and verbal fluency, are widely used as tests of frontal lobe control functions. Since the pioneering work of Shallice and Burgess (1991), it has been known that complex, naturalistic tasks can capture deficits that are missed in these classical tests. Matching this finding, deficits in several classical tasks are predicted by loss of fluid intelligence, linked to damage in a specific cortical “multiple-demand” (MD) network, while deficits in a more naturalistic task are not. To expand on these previous results, we examined the effect of focal brain lesions on three new tests–a modification of the previously-used Hotel task, a new test of task switching after extended delays, and a test of decision-making in imagined real-life scenarios. As potential predictors of impairment we measured volume of damage to *a priori* MD and default mode (DMN) networks, as well as cortical damage outside these networks. Deficits in the three new tasks were substantial, but were not explained by loss of fluid intelligence, or by volume of damage to either MD or DMN networks. Instead, deficits were associated with diverse lesions, and not strongly correlated with one another. The results confirm that naturalistic tasks capture cognitive deficits beyond those measured by fluid intelligence. We suggest, however, that these deficits may not arise from specific control operations required by complex behaviour. Instead, like everyday activities, complex tasks combine a rich variety of interacting cognitive components, bringing many opportunities for processing to be disturbed.

It has long been thought that complex, open-ended tasks may capture aspects of frontal “executive” impairment that are missed in the more constrained setting of conventional neuropsychological testing. In a pioneering study, Shallice and Burgess (1991) introduced two tasks designed to mimic the open-ended character of everyday problem solving. In the 6-element task, the patient had to divide a period of 15 min between six different tasks, with freedom to switch tasks whenever they chose, but with several additional rules concerning task order and time allocation. In the multiple-errands task, the patient undertook a list of activities in a street of shops, again organizing the entire performance to respect a list of rules and requirements. In three frontal patients, Shallice and Burgess (1991) showed major impairment in these tasks, despite generally good performance on a battery of more conventional executive tests such as Wisconsin card sorting (Milner, 1963), verbal fluency (Benton, 1968) and Trails B (Reitan, 1955).

In previous studies, we have investigated the link between executive tests and fluid intelligence, measured with a standard test such as the Culture Fair (Institute for Personality and Ability Testing, 1973). For a number of conventional tests, including card sorting, fluency, and Trails, deficits in several patient groups are largely explained by a loss of fluid intelligence; once fluid intelligence is partialled out, performance is largely equivalent for patients and controls (Roca et al. 2010, 2012, 2013, Roca et al., 2014). Fluid intelligence deficits are linked to damage in a distributed cortical “multiple-demand” or MD network, incorporating specific regions of lateral frontal, dorsomedial frontal, insular and parietal cortex (Woolgar et al., 2010, Woolgar, Duncan, Manes, & Fedorenko, 2018; Barbey et al., 2012; for evidence on white matter connections see Gläscher et al., 2010). Performance on tests such as card sorting, fluency, and Trails may largely reflect the functions of this network. Findings are different for a more open-ended task, the Hotel, designed to mimic the Shallice and Burgess (1991) 6-element task in a more realistic setting (Manly et al., 2002). For the Hotel task, we have repeatedly found that performance is only weakly related to fluid intelligence, and partialling fluid intelligence does not remove patient deficits (Roca et al. 2010, 2012, 2013, Roca et al., 2014). These findings suggest less specific dependence on MD functions.

In the present work, we extended these prior findings using three new tests, administered along with the Culture Fair to a group of patients with focal lesions in different regions of cortex. First, we used a version of the Hotel, somewhat shortened compared with previous versions. Second, we designed a new test of everyday problem-solving, based on short vignettes describing real-life situations and their accompanying decisions. Third, we designed a new task-switching test to mimic just one aspect of the complex processing requirements of the Hotel. It has often been suggested that, in this test, patients may fail to divide their time between component sub-tasks because, having become immersed in one, they forget the larger requirement to give some time to all of them (Manly et al., 2002). To investigate this kind of immersion as a possible key factor in naturalistic behaviour, we modified a standard task-switching paradigm (Rogers & Monsell, 1995) to manipulate the length of time before a task switch. Compared to our other two tasks, this one focused on a quite specific cognitive requirement, but one that we thought might be important in temporally extended, open-ended behaviour.

In line with our previous findings, we expected that deficits in fluid intelligence would be linked to MD lesions, but that neither fluid intelligence nor MD lesions would account for deficits in the three new tests. Instead we considered two hypotheses. First, we thought that naturalistic task deficits might follow from lesions to the brain's default-mode network (DMN). In functional brain imaging, the DMN is well known as a set of brain regions with strong functional connectivity (Yeo et al., 2011), deactivation in many tasks compared to rest (Shulman et al., 1997), but positive activation linked to mind-wandering (Mason et al., 2007; Christoff, Gordon, Smallwood, Smith, & Schooler, 2009), self-related thought (D'Argembeau et al., 2005), and sometimes large externally-directed task switches (Crittenden et al., 2015; Smith et al., 2018). As regards the Hotel, one specific reason for suspecting DMN involvement came from the previous results of Roca et al. (2010, 2011), who linked Hotel deficits to anterior frontal lesions. On the medial surface, anterior frontal cortex includes a large region of the DMN, and though Roca et al. (2011) combined lateral and medial patients, it seemed possible that the DMN component was responsible for Hotel deficits. More broadly, a large body of imaging work links the DMN to both social cognition and imagination of cognitive scenes or episodes (Frith & Frith, 2003; Addis et al., 2007, 2009; Amodio & Frith, 2006; Andrews-Hanna et al., 2010; Wen, Mitchell, & Duncan, 2020). Several authors have suggested that situation representations in the DMN place ongoing cognition in a broader context (Hassabis & Maguire, 2007; Ranganath & Ritchey, 2012; Smith et al., 2018). Reference to a larger context could be especially important in naturalistic problem-solving, including behavioural management over an extended time period as required in the Hotel, and our new test of decision-making in imagined, lifelike episodes. If naturalistic tests largely assess DMN function, we anticipated strong correlations between these tests in the patient sample, with deficits predicted by volume of damage to the DMN.

An alternative is that deficits in naturalistic tasks do not reflect the function of any specific brain network, and do not measure specific, identifiable control requirements brought into play for complex behaviour. Rich, varied tasks based on the requirements of everyday cognition are likely by definition to have many different cognitive components, dependent on multiple brain processes (Burgess et al., 2000). These components, furthermore, are unlikely to act independently; if one component is weakened through brain damage, it may send delayed or inaccurate input to others, or compete for mental resources. Of course, all tasks are potentially influenced by multiple sensory, motor and cognitive functions, but for rich tasks, it may be especially unlikely that one or a few core processes are responsible for the bulk of neuropsychological impairments. On this account, complex tasks are highly sensitive to brain damage simply because there are many opportunities for their processing to be disturbed. The prediction would be that there is no core deficit in “complex” behaviour, reflected in shared impairments across multiple tasks; and that deficits are not well explained by damage to any one cortical region or network.

## 1 Methods

### 1.1 Participants

A convenience sample of 34 patients was recruited from the Cambridge Cognitive Neuroscience Research Panel at the MRC Cognition and Brain Sciences Unit. The study was conducted in accordance with ethical approval from the Cambridge Psychology Research Ethics Committee. Patients were selected on the basis of having chronic, focal lesions from mixed aetiology excluding traumatic brain injury, and aged between 18-80 years old. There were no other formal exclusion criteria for region of lesion or specific cognitive deficit. Two patients were unable to complete more than one task and were excluded from further analysis. Demographic and lesion information for the remaining 32 patients is presented in Table 1. Thirty non-brain-damaged control participants were also selected, aiming for approximate match to patients in distributions of age and education. Accordingly, patient and control groups did not differ significantly in age (patients mean = 58.4 years, SD = 15.3; controls mean = 52.6, SD = 18.9; t(61) = 1.35, *p* = .18) or years of education (patients mean = 13.9, SD = 2.3; controls mean = 14.7, SD = 3.9; t(61) = 1.04, *p* = .30).

**Table 1:** Clinical and demographic data. For some patients precise lesion date is unknown and an estimate is given based on date of joining panel.

Patient	Age (years)	Years of Education	Aetiology	Lesion hemisphere	Time between lesion and tests (years)
1	73	11	stroke	right	11.5
2	32	16	stroke	right	8.5
3	47	11	tumour	right	1.5
4	74	11	tumour	right	>15
5	71	11	tumour	right	9
6	53	16	tumour	right	4
7	70	16	tumour	left	6.5
8	71	17	stroke	right	17
9	67	16	tumour	left	25.5
10	79	9	stroke	right	20
11	36	14	tumour	left	7
12	54	16	stroke	right	18
13	71	16	stroke	right	20
14	78	13	stroke	right	14
15	62	13	resection	right	19
16	54	14	abscess	left	13
17	68	11	tumour	bilateral	20
18	53	11	tumour	left	19
19	35	13	tumour	right	7.5
20	65	14	infection	left	12.5
21	42	16	tumour	left	9
22	54	11	tumour	right	14
23	30	13	tumour	left	3
24	47	13	tumour	right	13
25	73	17	tumour	left	3
26	60	13	tumour	left	19
27	77	18	tumour	right	13.5
28	25	14	tumour	right	5
29	46	16	tumour	bilateral	13
30	67	13	tumour	bilateral	8
31	64	16	tumour	left	18
32	67	16	unknown	right	>18

### 1.2 Testing

Participants were given a battery of computer-based and other tasks described below. The test battery was completed in a single session lasting around 90–120 min. The battery consisted of seven tasks, presented in fixed order. In this paper we present data for Culture Fair (presented first in the session), Situations (second), Hotel (fourth and seventh; see below), and Switch Time (sixth). The remaining three tests were included for other purposes, not concerned with naturalistic decision making. They were a more conventional task switching paradigm (presented third), adapted from Smith et al. (2018), an object in place concurrent discrimination memory task (presented fifth), similar to Gaffan (1994), and a comparison of attention control by scene or object cues (presented last). Four patients did not complete the Switch Time task due to fatigue. Additionally, for one of these four patients, the battery was split into two shorter sessions. Computer-based tasks were given on a Dell 1280 × 1024 resolution laptop, controlled using Psychophysics Toolbox for MATLAB (Brainard, 1997).

### 1.3 Tasks

#### 1.3.1 Culture Fair

Participants were given the standardised version of the Cattell Culture Fair, Scale 2 Form A (Institute for Personality and Ability Testing, 1973) consisting of four timed subtasks (series completion, odd one out, matrices, topological relations). At the start of each subtask, the experimenter read aloud the rules to the participant and went through three examples with them. Total correct scores were calculated and then converted into IQs from the standardised table of norms.

#### 1.3.2 Situations

The Situations task was designed to test social and non-social decision-making in real-life vignettes. During the task, participants were shown twelve short stories on the computer screen, and after each story asked one social judgement question, one emotion judgement question and one executive judgement question. The full set of stories and questions is presented in the Supplementary Materials. For each item, participants were first shown the story text and asked to read through the story. After reading, participants were asked to press a button. With the story text still present on the screen, the questions were then presented one by one, along with three possible answers each. The answer options were designed such that one was correct, one was very incorrect and the third was plausible but less optimal than the correct option. Participants were asked to respond as quickly as they could using buttons “1”, “2” or “3” on the keyboard, corresponding to the three possible answers. The order of story presentation was randomised and the order in which the questions were presented was pseudorandomised such that each type of question (social, emotional and executive) was presented equally often first, second and third. The position of the correct answer was counterbalanced across question types such that it appeared in positions 1, 2 and 3 on an equal number of trials.

We scored proportion error and median response time (RT) for correct trials. RTs for the three different question types were strongly correlated for both patients (for the three possible pairs of question types, r = .89 to .95) and controls (.49 to .71). Proportion error scores for the three question types were also correlated in patients (.22 to .59), though not in controls (−.03 to .11), perhaps in part because error proportions were low. As overall measures of performance, we averaged RTs and proportion error scores across the three question types.

#### 1.3.3 Hotel

The Hotel task used materials laid out on a table in front of the participant. Participants were asked to imagine they were in a job interview for a position at a hotel and were asked to try three different hotel activities, each one involving sorting a stack of sheets of paper. Participants were told that they would have 9 min to work on the three activities and that it would be impossible to finish any of them completely in the time limit. Instead, they should “try and do something from each task—spending as much time on each as possible within the total time available”. To keep track of the time participants were given a clock. Throughout the task the clock was turned away from the participants, but participants could choose to check it at any time before returning it to its backward-facing position.

Participants were given two variants of the task, which varied the form of periodic interruptions. The order of the two variants was counterbalanced across participants, with two other tasks performed between the two variants. Interruptions were motivated by the work of Manly et al. (2002), who found that performance improved when patients were given an occasional auditory alert designed to break focus on the current activity and reorient attention to the overall goal. In our version, interruptions were designed to reorient participants’ attention either within the current activity (internal interruption) or to the external environment (external interruption). In the internal interruption condition a yellow sheet of paper was placed after every seven task items in each activity. Participants were told to place the yellow item in the same pile as the previous item. In the external interruption condition a written instruction, asking participants to perform an action directed towards an aspect of the surrounding environment, was placed after every seven task items. Participants were asked to follow the written instruction (e.g., point to a window) and then place the instruction sheet to the side.

For the two task versions, there were two separate sets of three activities. The activity set paired with each interruption condition was counterbalanced across participants. Set A consisted of sorting conference name tags by alphabetical order, sorting invoices into piles according to the vendors, and sorting bills into piles according to customer name. Set B consisted of sorting staff name tags by alphabetical order, sorting restaurant lists into piles by their location, and sorting spa receipts into piles by treatment.

During the task, the experimenter kept a continuous record of which activity the participant was working on, using computer keys to indicate each time an interruption was encountered and each time the activity was switched, along with which new activity was begun. In line with previous work (Manly et al., 2002; Roca et al., 2010; Torralva et al., 2009), the score was the summed deviation from optimal time (180 s per activity) across the three activities. Preliminary analysis showed no differences between interruption types. However, there was evidence that participants (especially controls) improved their strategy when performing the task for the second time. Accordingly, for the main analyses, scores were based just on the first version performed, whichever interruption type it involved. In supplementary analyses we considered mean performance across the two task versions.

#### 1.3.4 Switch Time

One difference between the Hotel task and other classical switching tasks is the long period (∼180s) between switches. The Switch Time task was designed to test whether patients are particularly impaired at task switching after longer rather than shorter periods. As noted above, only 28 patients took part in this task. For each trial, participants were presented with a picture to the left of the screen and a word with a letter missing to the right of the screen. Participants were required to make yes/no judgements on one of these stimuli based on a task rule cued by a central shape, a blue square with either left or right corners slightly rounded. If the rounded corners of the cue pointed towards the left, then participants were asked to do the picture task. If the rounded corners of the cue pointed towards the right, then participants were asked to do the word task. The two tasks used were taken from Crittenden et al. (2015). For pictures, the decision was whether the item would fit in a shoebox; for words, it was whether addition of a letter ‘a’ in the blank position would create a real word. Participants were asked to make a “yes” or “no” response, by left or right keypress respectively. Performance was self-paced, with the stimuli remaining until a key was pressed, and participants were asked to respond as quickly as possible without making mistakes. An inter-trial interval (ITI) of 1.5 s followed each response.

Pilot studies revealed that switch performance systematically declined with increasing numbers of repeated trials prior to a cue change, reaching an asymptote at around twelve repeats. Accordingly, trials of the same task were repeated in blocks of four, six or twelve trials, with block length presented in a pseudorandom order. Trials could thus be sorted into four transition types: stay trials (task trials preceded by the same task), switch 4 trials (task switch after four of the same task), switch 6 trials (task switch after six of the same task), and switch 12 trials (task switch after twelve of the same task). The experiment consisted of one run of 182 trials. The run contained four blocks of each length for each task, plus a final run of six trials after the last switch. To provide a brief rest, a break was inserted into the run, approximately halfway through the trials, immediately after a switch trial. After the break, the task block continued with the same task cued as in the switch before the break. The last trial before the break and the first trial after the break were included for purposes of calculating run length before the next switch. This created a total of eight switch 4 trials, eight switch 6 trials, and eight switch 12 trials, with the remainder being repeat trials and the two start-up trials. The entire task lasted approximately 18 min.

Picture and word stimuli were centred 3.6 degrees of visual angle left and right of screen centre. All words measured approximately 5.6 (width) x 2.1 (height) degrees of visual angle. The pictures varied in proportions with maximum dimensions of approximately 6.0 (width) x 4.5 (height) degrees of visual angle. The cue was presented centrally measuring approximately 1.4 × 1.4 degrees of visual angle. To create the two cues, the left or right corners of a square were rounded using Adobe Photoshop. By using only a subtle difference between the two sides of the cue, we aimed to avoid a salient visual change that would alert participants to task switches.

There were equal numbers of “yes” and “no” trials for each combination of task and switch type. At the task switch trial and the first stay trial after a task switch, correct answers for the word task and the picture task were always different. For all other stay trials, the correct response for the cued task was randomised so that it was the same as the uncued task on half of the trials and different on the other half of the trials.

Before the start of the task, participants were shown two trials on paper and asked which task they would do given the cue shape and what button they would press in response to the cued stimulus. Participants continued with the main task only when the experimenter was satisfied that the participant understood the instructions. No subject needed more than two repetitions of the instructions before continuing with the main task.

Scores concerned proportion error and median correct RT on switch trials. To capture the effect of time since last switch, scores were proportion error on switch 12 trials minus proportion error on switch 4 trials, and similarly RT on switch 12 minus RT on switch 4.

### 1.4 Neuroradiological assessment

MRI T1 and T2 structural scans were acquired for all patients. Lesions were traced on structural images by a neurologist, blind to the experimental results, using MRIcron (Rorden & Brett, 2000) before normalising to MNI space using SPM software (Wellcome Department of Imaging Neuroscience, London, England; www.fil.ion.ucl.ac.uk) with cost-function masking to mask the lesion from the calculation of the normalization parameters (Brett et al., 2001).

### 1.5 Regions of interest

**Fig. 1:**
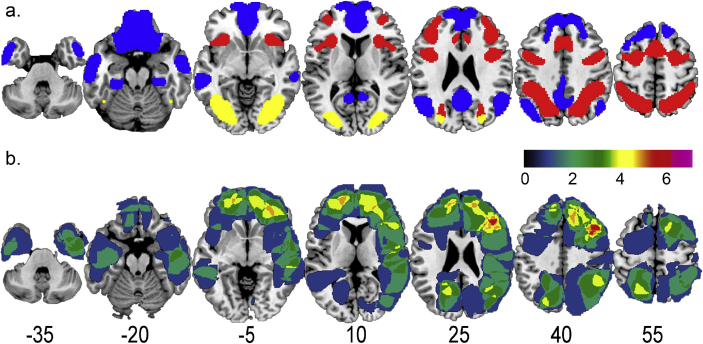
a. ROI volumes for frontoparietal MD network (red), DMN (blue) and the excluded occipital-temporal region (yellow). b. Patient lesion overlap. Colour bar shows number of patients. Numbers for each slice show z coordinate in MNI space. Left of brain is to left. Apparent extension of some lesions outside the brain reflects limitations of scan quality, tracing and/or normalization.

Volumes for the MD network, DMN and all other grey matter regions were constructed in the following steps. The resulting MD network (red) and DMN (blue) regions of interest (ROIs) are presented in Fig. 1a. Fig. 1b shows patient lesion overlap.

The MD ROI was based on data from Fedorenko, Duncan, & Kanwisher, 2013 (their Fig. 2), selecting only frontoparietal regions. This frontoparietal MD ROI (Fig. 1a, red) included the posterior–anterior extent of the inferior frontal sulcus, a posterior dorsal region of lateral prefrontal cortex, inferior frontal junction, anterior insula/frontal operculum, presupplementary motor area/dorsal anterior cingulate, and intraparietal sulcus. A template for these regions was downloaded from http://imaging.mrc-cbu.cam.ac.uk/imaging/MDsystem. These regions were combined to create one MD volume.

The DMN ROI, presented in blue in Fig. 1a, was generated using the three DMN networks from the liberal mask of the 17 network cortical parcellation reported in Yeo et al. (2011) (networks 15, 16 and 17). Additionally, network 10, containing temporopolar and orbital frontal regions was also included, as it contained ventromedial prefrontal cortex (vmPFC), a region often included in the DMN (Andrews-Hanna et al., 2010). These four networks were combined and smoothed with a 4 mm FWHM Gaussian smoothing kernel, and voxels with values > .5 after smoothing were retained.

As there was slight overlap between the insula/operculum component of the MD volume and the vmPFC component of the DMN volume, the region of overlap was removed from both ROIs.

An additional Other region was created using custom scripts for SPM 12 (Wellcome Department of Cognitive Neurology, London, UK). First a whole grey matter volume was created by concatenating all grey matter regions included in the AAL Atlas (Tzourio-Mazoyer et al., 2002). Then grey matter included in the MD or DMN ROIs, and all grey matter 5 mm or less from these volumes, was excluded. An additional occipital-temporal region is sometimes associated with the MD network but also strongly related to visual processing, and therefore this region (Fig. 1a, yellow) was also excluded. Remaining grey matter was assigned to the Other ROI. For each patient, volumes of damage were separately measured in MD, DMN and Other ROIs.

### 1.6 Preregistration, reporting and availability

No part of the study procedures or analysis plans was pre-registered prior to the research being undertaken. In the text, we report how we determined our sample size, all data exclusions, all inclusion/exclusion criteria, whether inclusion/exclusion criteria were established prior to data analysis, all manipulations, and all measures in the study. Data, digital stimulus materials and analysis code are available at https://osf.io/fyw2h/. The conditions of our ethics approval do not permit sharing of the raw MRI data with any individual outside the research team. Legal copyright restrictions prevent public archiving of the Cattell Culture Fair scale, which can be obtained from the copyright holder (https://www.hogrefe.com/uk/).

## 2 Results

### 2.1 Differences between patients and controls

Scores for each task are shown in Table 2 (left), separately for patients and controls. One-way analyses of variance (ANOVAs) showed that patients performed significantly worse than controls on all but one measure (Table 2, middle). To assess the role of fluid intelligence, we used analysis of covariance (ANCOVA) with Culture Fair IQ as a covariate. Results are shown in Table 2 (right), along with values of Pearson's r, derived from appropriate variance terms in the ANCOVA, reflecting the average within-group association between the variables. As predicted, though scores on two tasks (Hotel, Situations RT) were correlated with fluid intelligence, removing the effect of fluid intelligence left all significant group differences intact.

**Table 2:** Descriptive statistics and group differences for patients and controls.

	Patients		Controls		ANOVA			ANCOVA			
	*Mean*	*SD*	*Mean*	*SD*	*d.f.*	*F*	*P*	*d.f.*	*F*	*P*	*r*
Culture Fair (IQ)	96	22	107	15	(1,60)	5.07∗	.028	–	–	–	–
Hotel (time deviation, sec)	335	277	158	109	(1,60)	10.78∗	.002	(1,59)	6.53∗	.013	−.34∗
Situations (proportion errors)	.17	.08	.12	.05	(1,60)	7.47∗	.008	(1,59)	4.47∗	.039	−.26
Situations (RT, sec)	10.5	5.69	6.95	1.66	(1,60)	10.86∗	.002	(1,59)	6.15∗	.016	−.41∗
Switch Time 12–4 (proportion errors)	.08	.17	.02	.11	(1,56)	2.77	.101	(1,55)	3.29	.075	.12
Switch Time 12–4 (RT, sec)	.66	.91	.00	.36	(1,56)	13.27∗	<.001	(1,55)	11.57∗	.001	−.10

The table presents mean scores and standard deviations (SD) for patients and controls in the 6 task measures, followed by group differences between patients and controls before (ANOVA) and after (ANCOVA) accounting for differences in Culture Fair IQ. Asterisks (∗) represent significant effects (*p* < .05). d.f. = degrees of freedom, F = F-value, P = p-value, r = within-group Pearson's correlation with IQ.

As a direct comparison with Roca et al. (2010) these analyses were re-run for the 14 patients (12 for the Switch Time task) whose lesions were restricted to the frontal lobe. Results were similar to those obtained in the full group, except that, in the ANCOVA, the significant group difference for Situations, proportion error was removed.

For Switch Time, our primary scores concerned the effects of preceding block length on switch trials (difference between switch 12 and switch 4 trials). In this task, however, we note that patients also showed a substantial RT impairment even on stay trials (mean proportion errors .06 and .04 respectively for patients and controls, *p* < .08; median RT 2.40 and 1.57 s, *p* < .004).

### 2.2 Effects of lesion volume

The next analysis examined the relationship between behavioural scores and lesion volumes. This analysis was restricted just to the patient group. For MD, DMN and Other ROIs, the mean (standard deviation) volumes of damage were 8.39 (7.32), 8.43 (11.15) and 10.65 (7.76) ml respectively. Across patients, volumes of damage in the three ROIs were close to independent (maximum r = .14).

Following Woolgar et al., 2010, Woolgar, Duncan, Manes, & Fedorenko, 2018, we asked first whether Culture Fair IQ was predicted by volume of MD lesion. To account for multiple comparisons (ROIs), significance threshold for correlations was set to *p* < .017, one-tailed. Consistent with Woolgar et al., 2010, Woolgar, Duncan, Manes, & Fedorenko, 2018, patient IQ was found to be significantly related to MD lesion volume (r = −.53, *p* < .001). There was no relationship to DMN lesion volume (r = .21, *p* = .88), and only a weak association with Other lesion volume (r = −.35, *p* = .03). Total lesion volume was also not significantly related to IQ (r = −.19, *p* = .15).

As nine of the current patients were also tested in Woolgar et al., 2010, the correlation between Culture Fair IQ and MD lesion volume was re-calculated with those nine patients excluded. Results for this independent patient group replicated the Woolgar et al., 2010 findings (r = −.39, *p* = .03).

Similar analyses were then carried out for naturalistic task scores. The results are shown in Table 3. No measure was significantly related to lesion volume in either MD or DMN regions. Contrary to the DMN hypothesis, all correlations of naturalistic task scores with DMN lesion volume were close to zero, and in 3/5 cases negative. For the RT score (switch 12–4) from the Switch Time task, there was a significant correlation with lesion volume in the Other ROI. Naturalistic task measures were also unrelated to total lesion volume.

**Table 3:** Correlations (Pearson's r) between lesion volume within each ROI and naturalistic task performance.

	MD	DMN	Other
Hotel (time deviation, sec)	.23	−.06	.11
Situations (proportion errors)	.28	−.15	.11
Situations (RT, sec)	.27	.09	.33
Switch Time 12–4 (proportion errors)	−.15	−.12	−.22
Switch Time 12–4 (RT, sec)	−.19	.05	.56∗

Significant values (one-tailed, correcting for multiple comparisons across three ROIs) are shown with an asterisk (∗).

### 2.3 Between-task correlations

Our next set of analyses asked whether deficits in different naturalistic tasks were closely related to one another, suggesting some common cognitive component. To examine this, between-task correlations were performed separately in patient and control groups. Results are shown in Table 4, with patient group correlations in the top triangle and control group correlations in the bottom triangle. With only a single exception (patient group, correlation of Situations RT with Switch Time RT difference), correlations were uniformly nonsignificant.

**Table 4:** Between task correlations (Pearson's r) for patients (top triangle) and controls (bottom triangle).

	Hotel (time deviation, sec)	Situations (proportion errors)	Situations (RT, sec)	Switch Time 12–4 (proportion errors)	Switch Time 12–4 (RT, sec)
Hotel (time deviation, sec)		.23	.31	−.33	−.01
Situations (proportion errors)	−.08		.35	−.20	−.24
Situations (RT, sec)	.15	.03		.08	.43∗
Switch Time 12–4 (proportion errors)	.23	.36	−.15		−.21
Switch Time 12–4 (RT, sec)	.02	−.21	−.03	−.02	

Asterisks (∗) represent significant two-tailed correlations (*p* < .05).

### 2.4 Residual patient impairment

**Fig. 2:**
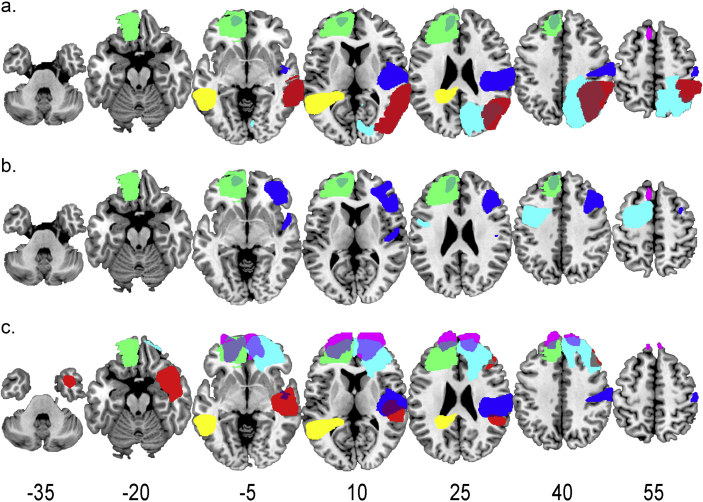
Lesion overlap for the patients with the greatest residual impairment after accounting for differences in Culture Fair IQ. a. Lesions of the six most impaired patients from the whole sample. b. Lesions of the four most impaired patients when the patient sample was restricted to patients with only lesions to the frontal lobe. c. Lesions of the six most impaired patients on Switch Time only. For each subplot, each colour denotes a different patient. Numbers for each slice show z coordinate in MNI space. Left of brain is to left.

As a further examination of lesion effects, we calculated average performance across naturalistic tasks, after accounting for effects of fluid intelligence. For each patient, standardized residual scores, calculated from the above ANCOVAs, were derived for all five naturalistic-task scores and then averaged. Signs were set such that high scores reflected poorer performance than predicted from Culture Fair IQ. For the four patients who had not completed the Switch Time task, the average residual score was generated from Hotel and Situations scores. After correcting for multiple comparisons, the average residual was not correlated with volume of damage in any ROI, with the strongest correlation for Other (r = .31). Fig. 2a shows the lesions of the six patients with the highest average residual scores, reflecting naturalistic task performance worse than predicted from fluid intelligence. Rather than implicating a specific brain region, the figure illustrates the diversity of lesions associated with naturalistic task impairment.

To compare with Roca et al. (2010, 2011) we recalculated patient residuals after restricting the patient sample to patients with frontal lesions only. Fig. 2b shows the lesions of the four frontal patients with the highest average residual scores. Unlike Roca et al. (2010), residual impairment after accounting for IQ was not restricted to the anterior PFC but spread across the frontal lobe. It should be noted, however, that this analysis used a small sample of 14 patients only.

As the Switch Time RT score was significantly related to damage in the Other ROI, we conducted a final similar analysis to search for a common lesion location among patients with the greatest deficits on this score only. The analysis was the same as for average residual scores (Fig. 2a), but this time using only the Switch Time residual. Again the results showed scattered brain lesions among the six patients with the greatest deficit (Fig. 2c).

### 2.5 Hotel: combined data

To confirm our results concerning the Hotel task, analyses were repeated using the average of the two task versions, instead of just the first. One patient who did not complete both versions was excluded. Again, there was a significant difference between patients and controls, F(1, 59) = 7.53, *p* < .01, not removed by covarying fluid intelligence, F(1, 58) = 4.41, *p* < .05. Performance was not significantly related to volume of lesion in MD, DMN or Other regions, r = .15, −.06, .15 respectively. Correlations with other naturalistic tasks were low in both the control group (median r = .09) and patients (median r = .08).

## 3 Discussion

In previous work, we have found that “executive” tasks vary widely in the degree to which deficits are explained by fluid intelligence (Roca et al., 2010, 2012; Woolgar et al., 2010). For several classical tests, such as Wisconsin card sorting, impairments in diverse patient groups are eliminated when fluid intelligence is partialled out. The Hotel task of Manly et al. (2002), however–based on the 6-element task of Shallice and Burgess (1991) – shows a very different result. For this task, patient impairments are not removed by partialling out fluid intelligence. These results match the long-held belief that complex, naturalistic tasks can reflect aspects of cognitive deficit that are missed in classical tests.

In the present work, we confirmed these findings for a new version of the Hotel task. Again, deficits in a diverse group of patients with focal brain lesions were not accounted for by fluid intelligence. Results were similar in a second test of understanding and decision-making in complex, life-like scenarios. In a third test, we attempted to isolate one critical factor in the Hotel task and potentially other real-world situations–the need to break out from a lengthy period of immersion in a single task. While task switching in the Hotel is spontaneous, with no external cue, we used an explicit switch cue but varied the length of the previous task block. Here too we observed deficits in the patient group that were not explained by fluid intelligence. Though fluid intelligence may account for deficits in many “executive” tasks, our results extend the list of more naturalistic tasks for which this is not true.

Also replicating previous work, we found that deficits in fluid intelligence were predicted by the extent of lesions to the frontoparietal MD network. MD lesions, however, were not strong predictors of deficits in the naturalistic tasks. While MD lesions may explain many aspects of classical executive deficit, again these data suggest a different explanation for deficits in more naturalistic tasks.

To explain naturalistic deficits, we considered two hypotheses. First, we thought it possible that such deficits arise largely from damage to the DMN, reflecting a broad role of this system in managing temporally-extended cognitive contexts, events or episodes (e.g., Ranganath & Ritchey, 2012; Andrews-Hanna et al., 2010). Plausibly, broad contextual representations will be especially important in naturalistic, open-ended behaviour. The data, however, tell strongly against this hypothesis. There was no suggestion that deficits were predicted by volume of damage to the DMN. Furthermore, deficits in our three naturalistic tasks were largely uncorrelated, suggesting no specific, common control process or processes.

Instead, we found that naturalistic deficits could arise from lesions scattered through multiple regions of the brain, in left or right hemispheres, in frontal, parietal or occipito-temporal cortex, and including or not sections of MD and DMN networks. Though performance was not significantly associated with either MD or DMN lesions, our interpretation is not that these networks make no contribution to naturalistic behaviour. Rather, no one network is strongly predictive of deficit simply because deficits can arise from lesions of many different kinds.

Our results for Hotel and Situations are firmly in line with the finding that complex, naturalistic tasks capture cognitive deficits missed in many executive tests, but suggest that these tests will be poor at isolating any specific cognitive process. In line with the arguments of Burgess et al. (2000), complex, real-life decision making rests on many processes, likely dependent on many different cortical regions. The particular processes that are critical may vary between tasks, matching our finding of generally nonsignificant correlations between them. Perhaps not surprisingly, complex, real-life decision-making may depend on much of the cortex, making these tests highly sensitive to brain lesions, but not highly diagnostic of any specific cognitive deficit.

For Switch Time this argument is less clear, since the test is ostensibly much more focused in its cognitive demands. Results however were similar, with deficits not explained by fluid intelligence, and unrelated to volume of damage in either MD or DMN regions. For this test there was a significant correlation with damage to the residual, Other ROI, but given widely scattered lesions in the most impaired patients, along with differences in total size of MD, DMN and Other regions this result should be interpreted with caution. Further work would be needed to analyse deficits in this task. By design, the cue indicating a change of task had low visual salience, perhaps requiring participants to maintain a sustained attentional set for its possible occurrence. The duration of a block of 12 trials depended on individual RTs, with typical durations around 40 sec for controls, but appreciably greater for patients given slow responses even on stay trials. The data suggest that escape from a period of immersion in one task indeed calls on cortical functions unlike those captured in fluid intelligence and, by extension, other classical executive tests. Again, however, deficits may have mixed causes, not simply related to specific lesion locations.

In principle, the source of a deficit in any one task and patient might be clarified by extensive neuropsychological testing. Given the low correlations between our naturalistic tasks, however, it seems unlikely that any small set of cognitive processes will be broadly predictive of “naturalistic-task” deficits. Instead, we suggest that each such task has its own, rich cognitive profile, likely impaired for different reasons in different patients.

On this interpretation, naturalistic tasks do not identify specific control deficits required only in complex, open-ended behaviour. Instead, their sensitivity to brain damage arises simply through the many cognitive processes that must be combined, bringing many opportunities for performance impairment. In line with the original work of Shallice and Burgess (1991), such tests may be especially useful as an indication of difficulties that patients may face in return to everyday life. To predict such difficulties, clinicians should use not only measures that target specific cognitive abilities, such as classical executive tasks which are associated with lesion to the MD system (Roca et al., 2010), but also tasks–like the ones described here–that tackle more complex, naturalistic scenarios. This way, we can provide a more comprehensive cognitive assessment which could better reflect real life deficits. Regarding neuropsychological rehabilitation, the findings shed light on the complicated task that neuropsychologists face when dealing with brain lesions. As clinical experience shows, difficulties in returning to everyday life are not easily predicted from damage to specific brain regions or networks, reflecting the complexity of cortical involvement in everyday behavioural management and decisions.

## CRediT author statement

Conceptualization–VS, CP, DJM, MR, JD; methodology–VS, CP, DJM, MR, JD; software–VS, DJM; formal analysis–VS, DJM; investigation–VS, CP, JA, TD; writing original draft–VS; writing review and editing–VS, CP, JA, DJM, TD, MR, JD; visualization–VS, DJM; supervision, project administration and funding acquisition–MR, JD.
