# Supplementary material for: Fluid intelligence and naturalistic task impairments after focal brain lesions
Source: Cortex. 2022 Jan;146:106–15. doi: 10.1016/j.cortex.2021.09.020 (PMC8769046; doi:10.1016/j.cortex.2021.09.020)
Supplement: Multimedia component 1 [file mmc1.docx]

Supplementary Material

Situations Task – Full Question Set

* = correct answer, ^ = second best answer.

Gabrielle has recently changed jobs and she´s going through a financial struggle. Her sister Amy´s birthday was coming up so she had been walking to work in order to save the bus fare to get her something pretty. She finally saved up a little and bought Amy a bracelet. The day of the birthday Gabrielle was at her sister´s house with some of Amy´s friends. After Amy opened all the presents, Gabrielle decided to start doing the dishes in order to help Amy out. She then heard Amy saying to her friends that she did not like the bracelet, because it looked cheap and tacky. After a few days the bills finally arrived, and Gabrielle noticed that she had spent more money on the bracelet than she should have. Now she didn’t have enough money to pay her bills.

*Executive question*

In order to pay her bills Gabrielle should:

1. Pawn her jewellery^
2. Rob a bank
3. Work out which bills can be put off until her next pay check*

*Feelings question*

When Gabrielle heard Amy she felt:

1. Hurt*
2. Perplexed^
3. Inspired

*Social question*

She should:

1. Trash Amy´s room
2. Wait until everybody has left and tell Amy how she felt*
3. Get angry at Amy and not speak to her for a while^

Josephine and David are getting married in November. They have agreed to a simple wedding in David´s parents’ garden, with a few close friends and relatives, because they are saving to buy a house in Canterbury. The garden is small, but they have planned to fit in four tables for the guests they have invited. They are eating out at a nice restaurant, while discussing the final arrangements of the wedding. Suddenly, Josephine receives an email from one of her college friends saying they heard about the wedding and five of them have booked flights to participate in it.

*Social question*

For the wedding, David should wear:

1. A dress
2. A dinner jacket^
3. A suit*

*Feelings question*

About this situation Josephine felt:

1. Nervous^
2. Concerned*
3. Rejected

*Executive question*

Regarding the space issue, Josephine should:

1. Try to fit in an extra table*
2. Cancel the wedding
3. Move the meal to a nearby park^

Nick was on holiday at an all-inclusive resort in Playa del Carmen, México. One beautiful and warm morning he decided to go for a walk to get to know the place a little better. The water was so clear, he could see both his feet and the fish swimming around them. As he walked by another resort, he happened to run across his boss´s boss, Colin. Colin recognized Nick, and asked him to join Colin and his young wife for dinner that night at one of the six restaurants in Colin’s hotel.

*Feelings question*

Regarding Colin’s invitation Nick felt:

1. Excited*
2. Worried^
3. Empty

*Executive question*

Nick did not remember the name of the restaurant. He should:

1. Go to MacDonald’s alone
2. Call the hotel and ask to speak to Colin’s room to ask him the name of the restaurant*
3. Go to every restaurant in the hotel and try to see if Colin is there^

*Social question*

When he meets Colin’s wife, Nick should be:

1. Friendly*
2. Flirtatious
3. Very Formal^

Michael´s son Trevor had been asking him to teach him how to drive for a very long time now. When Trevor turned 19 and got his first job Michael thought it was time and started teaching him with his brand new Toyota Corolla. Trevor was a very good driver, respectful and prudent. On their way back from Trevor’s driving test, Trevor asked Michael if he could drive the last block. Michael agreed and changed places with him. As soon as Trevor started driving a dog appeared, he tried to dodge it and crashed into his neighbor's car. Michael could not immediately afford the cost of repairs, and had been planning to drive friends to Edinburgh in two weeks' time.

*Executive question*

To deal with costs Michael should:

1. Borrow money for the repairs on his credit card*
2. Sell his car
3. Wait a few months until he can save enough money^

*Social question*

Michael should tell Trevor to:

1. Ring the neighbor's bell, tell him what happened, apologize and offer to pay for the damages*
2. Leave a note with the insurer’s details but not mention he was the driver^
3. Escape the scene

*Feelings question*

About the accident Trevor felt:

1. Loved
2. Anxious^
3. Ashamed*

Sonia has just started at a new job. She was working by herself as a party planner but got fed up and decided to try something different. As she has majored in marketing she applied for a position in a company and luckily got the job. She started on Monday and on Friday she was invited to a party at the house of one of her co-workers. The gathering was on the other side of town and it took her forever to get there. As she was arriving she looked for her phone to check the exact apartment when she noticed that it wasn´t there. She had left it charging in the bathroom! She was able to remember the address but not the apartment number.

*Feelings question*

Sonia felt:

1. Useless^
2. Angry with herself*
3. Jubilant

*Executive question*

Sonia should:

1. Ring one of the apartments and ask if they know her co-worker´s apartment number^
2. Go back home
3. Look around to see if someone else is arriving*

*Social question*

For the party, she should bring:

1. A nice bottle of wine*
2. A can of beer^
3. A Christmas pudding

Samantha has two sons, Jack aged 5 and Thomas aged 2. That afternoon she picked up Jack from school and took him to soccer practice. She took Thomas with her as well in order to take him to the park for a while. She was returning from the park on her way to pick up Jack from the football field, when she drove past a meat market and decided to stop to buy some meat for dinner. As soon as she got out, the car locked behind her with Thomas still inside. She tried everything she could think of but couldn´t open the doors and Thomas started crying. At the same time she knew Jack would be waiting on his own.

*Feelings question*

When the door locked Samantha felt:

1. Cheerful
2. Enraged^
3. Desperate*

*Social question*

To make arrangements for Jack she should:

1. Call her husband and ask him to pick Jack up*
2. Call the school and ask if a teacher will look after him^
3. Think it’s time for him to grow up anyway

*Executive question*

Samantha should:

1. Look for something to break a window^
2. Buy the meat and deal with this later
3. Find the number of a local locksmith*

Tim had been planning a trip to Salzburg for six months. He read a lot of reviews about where to go and what to eat. He flew on Wednesday to Salzburg, and arrived really early. Since the check in at the hotel started at 12.00am he decided to grab a bite in the meantime. He finally arrived at the hotel he had booked at 11.55am feeling very tired from walking around. Upon his arrival he was told at the front desk that they had no reservation under his name and no rooms available because of a convention happening that week. They offered to find him a room in another hotel, but this was much more expensive and completely beyond his budget.

*Social question*

Tim should:

1. Ask to speak to the manager and try to solve the situation*
2. Punch the receptionist
3. Shout at the receptionist and give the hotel a bad review^

*Executive question*

If there really is no room, Tim should:

1. Try to find another hotel at the right price*
2. Stay at the new hotel he was offered but cut his visit short^
3. Fly back home

*Feelings question*

Tim felt

1. Confused
2. Bored^
3. Annoyed*

Stella has a very good relationship with her mother-in-law, Frances. Frances really helps Stella with the kids, but she is also very talkative. Stella is at her work at the bank and she has a very busy day as a Director is visiting from France. Frances has been wanting to talk with her for days about what decorations she can buy for one of the children’s birthday parties, which is going to take place tomorrow. Stella is in the middle of an urgent report when she sees her mother-in-law calling her cellphone. She wants to take the call because the birthday is tomorrow but has no time to discuss the decoration right now. If she does not answer Frances, she will probably end up having to buy everything herself and having to bake the cake herself as well.

*Feelings question*

When Stella sees the call she feels:

1. Torn*
2. Proud
3. Uneasy^

*Social question*

Stella should:

1. Pick up the phone and tell her mother-in-law she won’t be able to talk right now*
2. Ignore her call and deal with her when she gets out of work^
3. Quit her job

*Executive question*

If she can’t get Frances’ help, Stella should:

1. Have no decorations for the birthday party^
2. Cancel the birthday party
3. Buy the decorations and a prepared cake from a shop*

Greg and Scarlett had been planning a trip to Thailand for a long while. They thought and took care of every little detail: flights, hotels, tours, transportation, they even arranged with Greg´s father to take care of Rocko, their dog, while they were away. A day before their trip, Greg´s father called them and told them he had decided to go on a last minute trip and wouldn´t be able to take care of Rocko. They called a few relatives and friends about looking after the dog, but without success.

*Social question*

Responding to his father, Greg should:

1. Get mad at his dad^
2. See if there is any way he can postpone his trip*
3. Break down in tears

*Feelings question*

Talking to his friends, Greg felt:

1. Frustrated*
2. Disconcerted^
3. Fulfilled

*Executive question*

To deal with Rocko they should:

1. Postpone the trip^
2. Put Rocko in a kennel*
3. Give the dog away

Benjamin’s wife is having a surprise birthday party for her sister. She has asked Benjamin to pick up the cake and present she ordered, because the stores are really close to his office. She advised him to be on time because the stores close at 5pm. Benjamin gets caught up with work and forgets about his errand. On his way out of the office at 5.30pm he realizes he has not bought what he was asked for. Instead he buys pudding in a nearby store and arrives to the birthday party late, with no cake or present.

*Executive question*

Benjamin should have:

1. Used his lunch break to buy all the things in advance*
2. Skipped the party and gone golfing
3. Set an alarm for 4.50 to go to the shops^

*Social question*

When he gets to the party Benjamin should:

1. Apologize to his wife and sister-in-law for what has happened*
2. Have a nap in his car and then go in
3. Say they should have fun anyway^

*Feelings question*

Benjamin’s wife feels:

1. Ashamed^
2. Safe
3. Angry*

Charlie and Peter went to see a movie. As Peter had an early dinner they caught the last show. As they were getting out of the cinema, Charlie asked Peter if he could wait while he went to the restroom. Peter was very tired so he declined, said goodbye and went his own way. Charlie went into the bathroom and when he tried to get out he realized the door was locked. He shouted, but nobody responded. It was very late and there were few people left at the cinema.

*Executive question*

To resolve this situation he should:

1. Sit and wait until someone appears^
2. Continue shouting and knocking on the door*
3. Smash the window and try to jump down into the street

*Feelings question*

Charlie felt:

1. Perplexed^
2. Frustrated*
3. Alive

*Social question*

When Charlie finally escapes he should:

1. Write a letter to the cinema explaining what happened and ask for an apology*
2. Never go to that cinema again^
3. Set the cinema on fire

George is on his way to work, where he has an important meeting with a potential client. He lives an hour away from his work and really enjoys driving there every day, because he drives through really beautiful places. As he has almost reached the city center, he realizes he has forgotten his laptop with the presentation at home. If he goes back to get it he will be very late for the meeting. At that moment his wife calls him and tells him she noticed he had left behind his laptop. She is already in the car bringing it to him, but she will get there probably half an hour after the meeting has started.

*Social question*

Because he doesn’t have his presentation with him George should:

1. Explain what happened and ask to move the meeting half an hour*
2. Make up an excuse (e.g. car problems) and wait until his wife arrives^
3. Buy the client a present

*Executive question*

To make sure this never happens again, George should:

1. Always leave his laptop with his car keys*
2. Move house so he is closer to work
3. Always arrive for work very early^

*Feelings question*

Speaking to his wife, George feels:

1. Delighted^
2. Grateful*
3. Sad
